# A Rare Case of Large Bowel Obstruction Secondary to Bone Grafting

**DOI:** 10.7759/cureus.8700

**Published:** 2020-06-19

**Authors:** Sri Hari Priya Vemulakonda, Angeline Mary Samy, Prashanth Venkatesan Venkatesan, TP Elamurugan, Nanda Maroju

**Affiliations:** 1 General Surgery, Jawaharlal Institute of Postgraduate Medical Education and Research, Puducherry, IND; 2 Surgery, Jawaharlal Institute of Postgraduate Medical Education and Research, Puducherry, IND

**Keywords:** incisional hernia, autologous iliac bone grafting, intestinal obstruction

## Abstract

Anterior abdominal wall incisional hernias can occasionally present as acute intestinal obstruction. Incisional hernias occurring at uncommon sites or after uncommon surgeries may contribute to diagnostic dilemmas. Herein, we report the case of a 53-year-old lady who presented with obstructed incisional hernia following autologous iliac bone grafting. We report this as a rare case of obstructed incisional hernia following an orthopedic procedure.

## Introduction

Intestinal obstruction is a common surgical emergency, usually secondary to postoperative adhesions (60%), malignancy (20%), hernia (10%), Crohn’s disease (5%), and miscellaneous causes (<5%) [1].

Hernias are the third leading cause of intestinal obstruction, and commonly occur in the ventral and inguinal regions. Lumbar hernias, which are relatively rare, are mostly acquired (80%) [1]. Primary acquired lumbar hernias occur spontaneously through the superior and inferior lumbar triangles. Secondary acquired lumbar hernias follow trauma or lumbar surgical procedures.

Iliac bone harvesting surgery is a common orthopedic procedure. Herniation of the intra-abdominal contents through the resulting bone defect is an extremely rare complication [2].

## Case presentation

A 53-year-old, otherwise healthy lady presented to our emergency department with a two-day history of colicky abdominal pain, associated with non-bilious vomiting, abdominal distension, and obstipation. Her past medical history was unremarkable except for autogenous left iliac bone grafting performed for lumbar spondylolisthesis 27 years prior. Clinically, she was hemodynamically stable. Her abdomen was distended, with exaggerated bowel sounds. Healed surgical scars were present over the left iliac crest and posterior midline. Examination of the abdominal wall and hernial orifices was normal.

**Figure 1:**
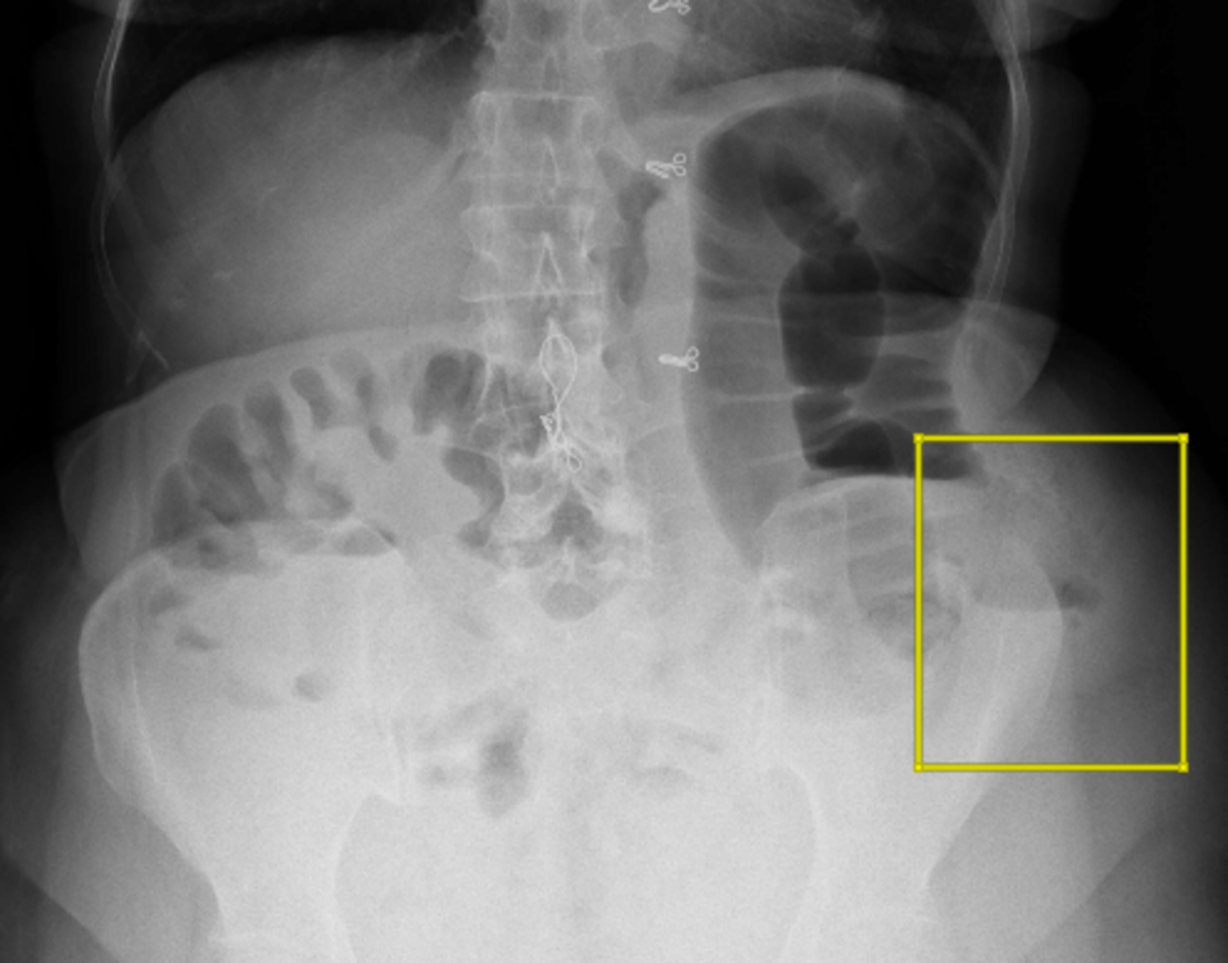
Plain radiogram shows dilated bowel loops with air-fluid levels near the left iliac region (inside the square).

Plain abdominal radiographs revealed dilated bowel loops and multiple air-fluid levels in the left-lower quadrant (Figure 1).

**Figure 2:**
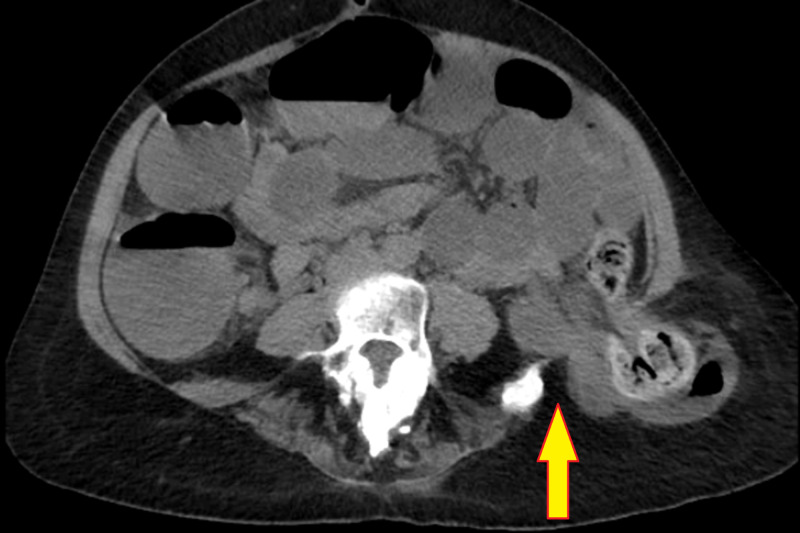
Contrast-enhanced CT shows the herniated colon (arrow) through the iliac bone defect, and a fecalith in the herniated colon.

**Figure 3:**
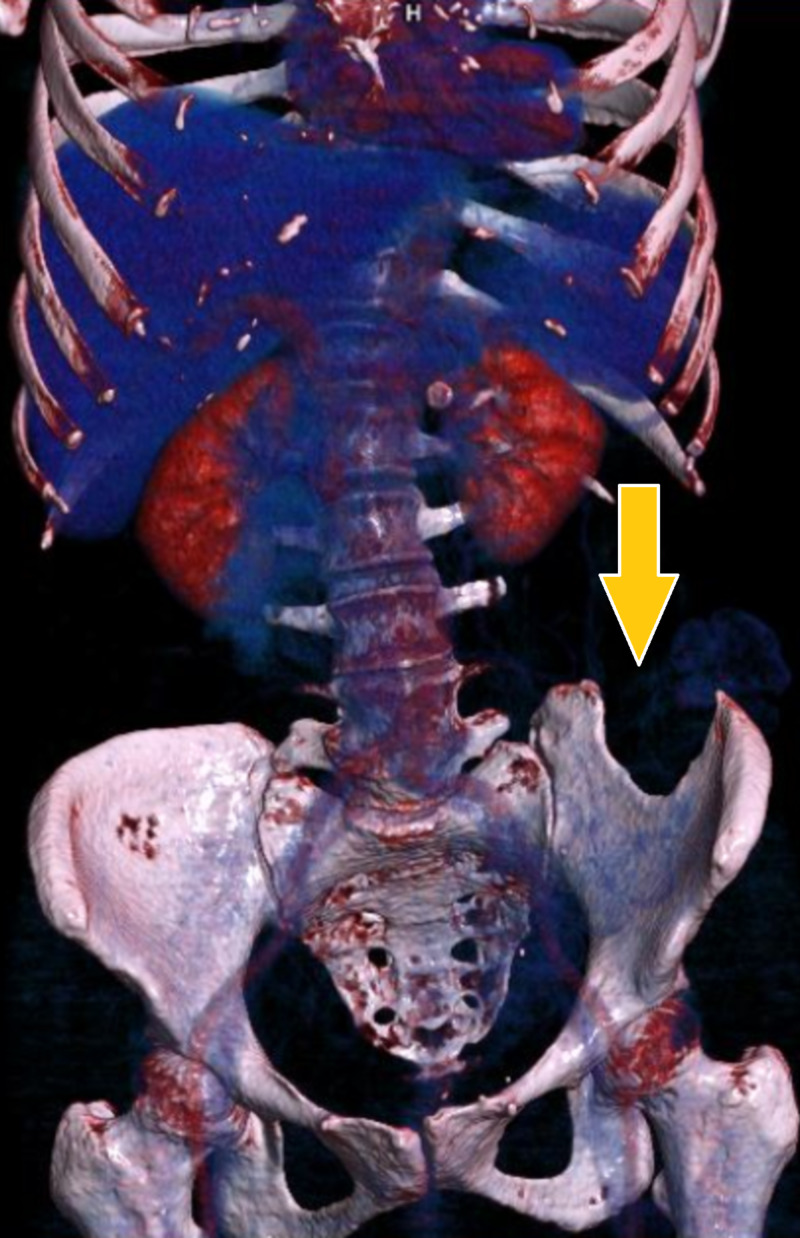
3D reconstructed contrast-enhanced CT image showing the defect in left iliac bone (arrow).

Abdominal contrast-enhanced CT (CECT) revealed a bony defect in the left iliac crest of size 3.3 cm, with descending colon and small bowel loops herniating through the defect. A large fecalith was also identified in the herniated colon. Transverse colon, ascending colon, and small bowel loops were dilated with the transition point being present at sigmoid colon. There was no evidence suggestive of ischemic changes in the entrapped bowel (Figures 2, 3). 

**Figure 4:**
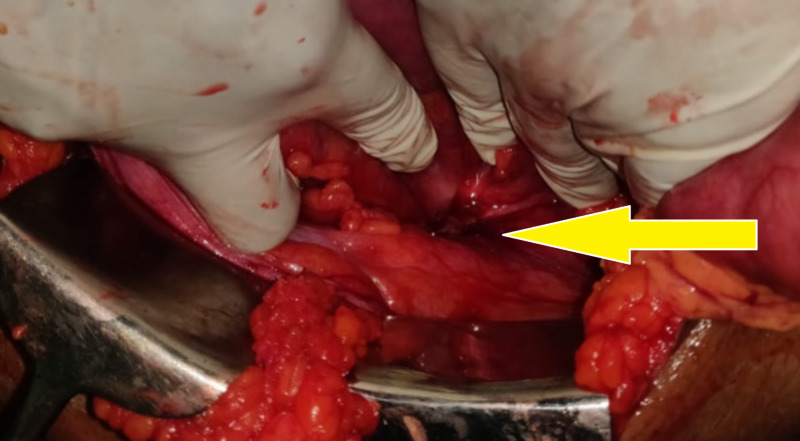
Intraop image of the defect along with herniated bowel (arrow).

At emergency surgery, a defect of size 3 × 3 cm was present in the iliac crest region. A loop of sigmoid colon with fecalith was found herniating through the defect (Figure 4). The defect was enlarged dividing the adjacent muscular layer superiorly to facilitate reduction of the herniated bowel, which fortunately was viable. The defect was closed with a preperitoneal polypropylene mesh fixed to the periosteum of the iliac bone inferiorly, and the muscular layer superiorly. She recovered rapidly following surgery and was discharged on postoperative day 5. On three-month follow-up, she was asymptomatic and had no evidence of recurrence.

On probing further, the lady explained that she was aware of intermittent distension in her left flank, associated with constipation, and that she used to manipulate the flank area for relief of discomfort as well as constipation. 

## Discussion

Autogenous iliac bone graft harvest is the gold standard for the treatment of bone defects, and hence frequently harvested. Several complications such as neurovascular injuries, ureteral injury, ileus, hematoma, fractures, and pelvic instability have been reported following this procedure.

Although rare, gastrointestinal herniation through the iliac bone defect is not unknown [2,3]. These cases usually present with incarceration (25%) or strangulation (10%) [4]. The first case was reported in 1945 by Oldfield in which he described the herniation of cecum [5]. About 40 cases have been reported so far. The incidence reportedly varies between 5% and 9%, and is common among females, older individuals (>65 years), malnourished, obese patients, and in cases where full thickness graft is harvested from the middle portion of iliac crest [3].

Abdominal wall lesions and herniation of abdominal organs at the ilium can be seen after tricortical graft harvesting [4]. The full thickness iliac crest fibro-osseous defect acts as a rigid ring through which abdominal contents can herniate. The hernial sac may contain retroperitoneal fat, kidney, colon, or less commonly small bowel, omentum, ovary, spleen, appendix, or liver [2,6]. In our case, the hernial sac contents were descending and sigmoid colon.

The onset of symptoms occurs from 24 days to up to 15 years following the iliac bone grafting surgery [2]. The usual presentation of these hernias is as chronic pain with or without a visible swelling. This may be associated with nausea, vomiting, and abdominal discomfort with or without a visible lumbar hernia. The history we received from our patient suggests that she was indeed symptomatic, although it did not precipitate an emergency anytime in the 27 years following surgery. This period of 27 years is longest in the published literature.

Clinical diagnosis is relatively simple if there is a mass in that region. In our case, the patient neither had chronic pain nor a swelling in that region. Large bowel obstruction was the index presentation of our case.

Lateral or oblique radiographs may show the bowel lying outside the abdominal cavity and ultrasonography may fail to demonstrate hernia due to low suspicion and presence of fat, both of which are not definitive for diagnosis. CECT of the abdomen is the investigation of choice, which delineates the muscular and fascial layers and outlines the defect and hernial sac [7].

Main treatment is surgery to reduce the hernia and repair the defect. The hernia can be approached by laparoscopy, retroperitoneal or transabdominal approach [8]. The hernial defect in our case was narrow, and a fecalith had formed in the herniated bowel, which made the reduction difficult. We had to cut open the muscle layer superiorly to deliver the bowel. The muscle layer was closed primarily and a tension free pre peritoneal mesh repair was done with polypropylene mesh.

Other treatment modalities would be advancement of soft tissues, such as abdominal muscles and fascia to bridge the gap, or a Bosworth technique, i.e., to change the profile of iliac crest by resection of bone and transposition of anterior superior iliac spine (ASIS) to a more distal and posterior portion [8].

Finally, the question remains whether this complication can be avoided while performing bone grafting. Bicortical bone grafts should be preferred to tricortical bone grafts to avoid a defect in this area. The inner and middle table of the iliac crest should be preserved. Care should be taken to avoid injuring the peritoneum while performing graft harvest. Other technologies like bone substitutes and cell therapy may also be considered [3].

## Conclusions

Herniation of abdominal contents secondary to iliac bone grafting is a rare complication, and hence results in a diagnostic dilemma, especially in the absence of typical symptoms. The diagnosis is often clinched by CECT. Prompt surgical correction is likely to result in favorable outcomes. 

## References

[1] Schnüriger B, Barmparas G, Branco BC, Lustenberger T, Inaba K, Demetriades D (2011). Prevention of postoperative peritoneal adhesions: a review of the literature. Am J Surg.

[2] d'Hondt S, Soysal S, Kirchhoff P, Oertli D, Heizmann O (2011). Small bowel obstruction caused by an incarcerated hernia after iliac crest bone harvest. ISRN Surg.

[3] Prabhu R, Kumar N, Shenoy R (2013). Iliac crest bone graft donor site hernia: not so uncommon. BMJ Case Rep.

[4] Kaushik R, Attri AK (2003). Incisional hernia from iliac bone grafting site—a report of two cases. Hernia.

[5] Oldfield M (1945). Iliac hernia after bone-grafting. Int J Res Orthop.

[6] Nodarian T, Sariali E, Khiami F, Pascal-Mousselard H, Catonné Y (2010). Iliac crest bone graft harvesting complications: a case of liver herniation. Orthop Traumatol Surg Res.

[7] Kane VG, Silverstein GS (1986). CT demonstration of hernia through an iliac crest defect. J Comput Assist Tomogr.

[8] Bosworth DM (1955). Repair of herniae through iliac-crest defects. J Bone Joint Surg Am.

